# hnRNPH1: A Multifaceted Regulator in RNA Processing and Disease Pathogenesis

**DOI:** 10.3390/ijms26115159

**Published:** 2025-05-28

**Authors:** Lijing Zhu, Wei Yi, Like Zhang, Chenyue Qiu, Ning Sun, Jingwen He, Ping Feng, Qiong Wu, Guangyi Wang, Guosheng Wu

**Affiliations:** Department of Burn Surgery, Changhai Hospital, Navy Medical University, Shanghai 200433, China; zhulijing@smmu.edu.cn (L.Z.); 13973140696@163.com (W.Y.); ssrliker@163.com (L.Z.); yue31109@163.com (C.Q.); 18845801850@163.com (N.S.); 19973599665@163.com (J.H.); 13564557674@163.com (P.F.); xiarizhiqun@163.com (Q.W.)

**Keywords:** hnRNPH1, RNA-binding protein, alternative splicing, pathogenesis

## Abstract

Heterogeneous nuclear ribonucleoprotein H1 (hnRNPH1) is a multifunctional RNA-binding protein (RBP) that plays a central role in post-transcriptional regulation. Through its quasi-RNA recognition motifs and low-complexity domains, hnRNPH1 specifically binds guanine-rich RNA sequences, including G-quadruplex structures, to precisely modulate multiple aspects of RNA metabolism, such as alternative splicing, mRNA stability, translation, and subcellular localization. Accumulating evidence has implicated hnRNPH1 dysfunction in the pathogenesis of several human diseases. In cancer, hnRNPH1 often acts as a pro-tumorigenic factor, albeit in a context-dependent manner, influencing the alternative splicing of crucial oncogenes, mRNA stability, and tumor cell sensitivity to therapeutic agents. In the nervous system, hnRNPH1 is involved in neurodevelopment, neurodegenerative diseases, and drug addiction and plays an essential role in maintaining neuronal function and homeostasis. Furthermore, it exerts regulatory functions in reproductive system development and fertility and in non-neoplastic pathologies, including cardiovascular diseases, autoimmune disorders, and viral hepatitis. Given its pathophysiological significance, hnRNPH1 has emerged as a promising biomarker and therapeutic target. This review provides an overview of the structural basis and core molecular function of hnRNPH1. Its mechanisms of action and pathological significance in various diseases have also been detailed. Additionally, this review summarizes the current therapeutic strategies targeting hnRNPH1, discusses the associated challenges, outlines optimization approaches, and considers future research directions. Overall, this review aims to deepen our understanding of hnRNPH1 biology and inspire the development of novel diagnostic and therapeutic interventions.

## 1. Introduction

Gene expression is a complex process regulated at multiple levels. Post-transcriptional regulation, particularly RNA processing and metabolism, plays a crucial role in determining cell fate and maintaining cellular homeostasis [1,2,3,4]. RNA-binding proteins (RBPs) play a pivotal role in gene expression [5,6,7]. By recognizing and binding to their target RNA molecules, RBPs precisely regulate multiple aspects of RNA fate, including splicing, stability, localization, and translation [8,9,10]. Among RBPs, the heterogeneous nuclear ribonucleoprotein (hnRNP) family is a widely studied and functionally diverse class involved in nearly every step of gene expression, from RNA transcription to protein synthesis [11,12,13].

Within the hnRNP family, heterogeneous nuclear ribonucleoprotein H1 (hnRNPH1) has attracted significant attention in recent years because of its critical functions. hnRNPH1 specifically binds guanine-rich (G-rich) RNA sequences via its distinct quasi-RNA recognition motifs (qRRMs) and low-complexity domains (LCDs) [14,15,16,17,18]. It also plays a key role in regulating alternative splicing (AS), mRNA stability, and translation [14,15,16,17,18]. These diverse molecular functions establish hnRNPH1 as a unique and critical node in the gene expression regulatory network.

Accumulating evidence has indicated that hnRNPH1 dysfunction is closely associated with the pathogenesis of several human diseases; its impact spans multiple pathologies. hnRNPH1 often plays complex, pro-tumorigenic roles in various cancers; is crucial for neurodevelopment and is implicated in neurodegenerative diseases and addiction; affects reproductive system development and function; and is involved in cardiovascular diseases, autoimmune disorders, and other pathological processes [19,20,21,22]. The tissue-specific and context-dependent nature of hnRNPH1 functions underscores their complexity and the importance of further investigation of their biology [19,23,24,25].

Given the broad impact of hnRNPH1 on developmental biology and disease pathophysiology, coupled with its emergence as a potential therapeutic target, this review aims to comprehensively summarize the current understanding and research progress concerning hnRNPH1. Here, we systematically discussed the structural basis and core molecular functions of hnRNPH1, and elucidated its mechanisms and pathological significance in cancer, neurological disorders, reproductive system disorders, and other non-oncological diseases. Furthermore, we summarized the current therapeutic strategies targeting hnRNPH1, associated challenges and optimization approaches, and concluded with future research perspectives. By integrating the current knowledge on hnRNPH1, this review seeks to offer a deeper understanding of the biological functions of hnRNPH1 and its multifaceted role in human diseases, thereby providing insights for the development of novel diagnostic and therapeutic strategies.

## 2. Structural Basis and Molecular Functions of hnRNPH1

### 2.1. Structural Architecture

**Figure 1:**
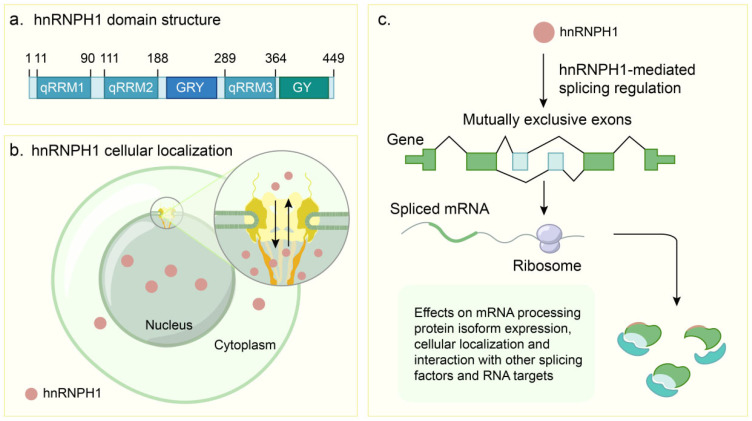
Domain architecture, subcellular distribution, and splicing regulatory mechanisms of hnRNPH1. (**a**). Schematic of the hnRNPH1 domain structure, featuring three quasi-RNA recognition motifs (qRRMs), a glycine-tyrosine-arginine-rich (GYR) domain, and a C-terminal glycine-rich (GY) domain. Numbers denote amino acid positions. (**b**). Subcellular localization of hnRNPH1. The protein resides primarily within the nucleus but possesses the capacity for nucleocytoplasmic transport. (**c**). Mechanism of hnRNPH1-mediated splicing regulation. hnRNPH1 interacts with specific RNA sequences, thereby influencing the selection of mutually exclusive exons during pre-mRNA splicing. This process impacts mRNA processing, protein isoform expression, subcellular localization, and interactions with other splicing factors and target RNAs. Subsequent translation of the spliced mRNA by ribosomes yields distinct protein isoforms exhibiting varied functions or subcellular distributions.

The *HNRNPH1* gene (located at 5q35.3) encodes a 449-amino acid nuclear protein (approximately 50–55 kDa) belonging to the hnRNP family. This protein plays a crucial role in RNA metabolism and gene expression regulation [26,27]. Its structure comprises multiple functional domains, including qRRMs, LCDs, and nuclear localization signals (NLSs), as shown in Table 1. These domains confer hnRNPH1 its unique RNA-binding properties and functional specificity. The domain architecture of hnRNPH1, including its key functional regions, is schematically illustrated in Figure 1a.

The core functional modules of hnRNPH1 are three qRRMs (qRRM1, qRRM2, and qRRM3) that mediate RNA binding [28]. Unlike typical RRMs, qRRMs lack key aromatic residues typically involved in RNA binding but retain the classic RRM fold [28,29]. qRRMs bind guanine (G)-rich RNA sequences, thereby modulating RNA secondary structures, particularly G-quadruplexes (G4) [28,30]. qRRM1 (located at the N-terminus) adopts a classical RRM fold and binds G-rich sequences, such as those found in exon 8 of the *EWSR1* pre-mRNA [31]. qRRM2 (located after qRRM1) contains a characteristic RGLP motif capable of resolving G4 s [26,27] tructures within oncogenic transcripts, like *HER2*Δ16 mRNA [14]. qRRM3 (located after qRRM2) also features a classical RRM fold and is involved in RNA binding and regulating RNA processing, including 3′-end processing by interacting with G-rich sequences/G4s and alternative splicing [27]. Notably, the C-terminal half of hnRNPH1, encompassing qRRM3 and the GY domain, exhibits transcriptional activation capacity when present in fusion proteins, as observed in B-cell precursor acute lymphoblastic leukemia (BCP-ALL) [14].

hnRNPH1 also contains a glycine-tyrosine-arginine-rich region (GYR domain) and a C-terminal glycine-rich region (GY domain). These LCDs play a significant role in mediating multivalent protein–protein and protein–RNA interactions (PRIs). The LC1 domain of hnRNPH1, which largely encompasses the GYR domain (located between qRRM2 and qRRM3), drives liquid–liquid phase separation (LLPS) [14]. LLPS is a fundamental biological process where proteins and nucleic acids condense to form dynamic, membrane-less compartments or granules within the cell [32,33]. These condensates are crucial for concentrating specific molecules and regulating various cellular activities. The phosphorylation of Tyr297 within this domain dynamically regulates stress granule assembly in response to cellular stress, such as mitochondrial retrograde signaling [34]. Disruption of the GYR domain’s LLPS capability, including mutations affecting Tyr297 phosphorylation, impairs the dynamic assembly of stress granules and leads to defects in mitochondrial homeostasis regulation [34]. Located at the C-terminus, the GY domain is notable for not forming hydrogel droplets in vitro [14,34]. The NLS of hnRNPH1 is located near the GYR domain and comprises a conserved YDPP motif and an adjacent basic region (amino acids 205–213) [35,36]. These elements are critical for the nuclear localization of hnRNPH1, where mutations or deletions result in nuclear and cytoplasmic localizations [35,37]. A schematic representation of hnRNPH1′s subcellular localization is shown in Figure 1b.

**Table 1:** Domain organization and functional characteristics of hnRNPH1.

Domain	Position	Structural Hallmarks	Binding Specificity	Core Functions
qRRM1	N-terminus (Approx. aa 11–90)	Classical RRM fold (β/α, β/β, α/β). Proline linker affects dynamics.	G-rich sequences, G-tracts, potential G4 structures.	RNA binding. Regulation of RNA processing, alternative splicing [28,31].
qRRM2	After qRRM1 (Approx. aa 111–188)	Classical RRM fold (β/α, β/β, α/β). Proline linker affects dynamics. Characteristic RGLP motif.	G-rich sequences, G-tracts, potential G4 structures.	RNA binding. Regulation of RNA processing, alternative splicing [27,38].
GYR	Between qRRM2 and qRRM3 (Approx. aa 190–260 region). Overlapping NLS region.	Low-complexity domain (LCD). Gly/Tyr/Arg-rich. Forms hydrogel in vitro. Phase separation driver.	Interacts with other LCD proteins.	Drives phase separation. Protein–protein interactions. Essential for physiological splicing function [14,34].
qRRM3	After qRRM2 (Approx. aa 289–364)	Classical RRM fold (β/α, β/β, α/β).	G-rich sequences, G-tracts, potential G4 structures.	RNA binding. Regulation of RNA processing (splicing, 3′ end). Involved in cancer fusion proteins [14,27].
NLS	Close to/overlapping GYR (Approx. aa 205–213)	Putative nuclear localization signal. Includes YDPP motif.	Recognized by karyopherin receptor complex (transportin).	Mediates nuclear import/shuttling. Mutations affect localization [35,36].
GY	C-terminus (Approx. aa 340–449)	Low-complexity domain (LCD). Glycine-rich. Does not form hydrogel in vitro.	Undefined RNA binding.	Can activate transcription in assays. Component of cancer fusion proteins [14,34].

### 2.2. Core Molecular Mechanisms of hnRNPH1

As a key RBP, hnRNPH1 plays a central role in regulating AS through various sophisticated molecular mechanisms. One core regulatory mode involves the direct recognition and binding of specific *cis*-acting elements on precursor messenger RNAs (pre-mRNAs), particularly G-rich motifs [39,40]. This binding typically occurs within the exonic or intronic regions of pre-mRNAs and can directly influence the selection of adjacent splice sites, thereby enabling bidirectional control over exon inclusion or skipping. For instance, hnRNPH1 can bind to G-rich motifs within “poison exons”, promoting their exclusion (skipping). This prevents the resulting mRNA from entering the nonsense-mediated decay (NMD) pathway and consequently upregulates the expression of certain genes, such as *RBM3* [39]. Conversely, hnRNPH1 promotes cassette exon inclusion by binding to intronic splicing enhancer elements, such as downstream G-rich sequences, as observed in the *MYC*-regulated *HRAS* gene [40]. The ability of hnRNPH1 to bind directly to target pre-mRNAs has been validated in diverse biological contexts, including its direct interaction with *SPO11* pre-mRNA in purified germ cells from wild-type (WT) mouse testes [19]. Beyond recognizing linear sequences, hnRNPH1 may also modulate splicing events directly or indirectly by influencing or resolving RNA secondary structures, such as G4 [28]. Although G-rich motifs are widely considered hnRNPH1 binding sites, their precise sequence specificity and RNA recognition patterns, compared to other RBPs with well-defined motifs, such as the CAAGR motif for FgRbp1 or the AU-rich hexanucleotide for CPSF30, require further in-depth investigation for complete elucidation [19,41,42].

In addition to direct RNA interactions, another crucial mechanism involves hnRNPH1, which acts as a hub for protein interactions. It indirectly influences the AS process by recruiting or synergizing with other splicing factors to form complex regulatory networks. This recruitment activity is critical for specific cell types. For example, in mouse germ cells (primary spermatocytes, round spermatids, and oocytes isolated from mouse tissues), the highly expressed hnRNPH1 recruits PTBP2 and SRSF3 to form regulatory complexes. These complexes collaboratively orchestrate a series of AS events essential for normal germ cell development (e.g., meiosis) and function (e.g., communication with supporting Sertoli cells) [19]. The specific knockout of *Hnrnph1* in germ cells resulted in widespread aberrant splicing, underscoring the importance of hnRNPH1 as a splicing coordinator [19]. In mammalian germ cells, the interaction network of hnRNPH1 extends beyond PTBP2 and SRSF3; it also interacts with BCAS2 and SRSF3 to promote the inclusion of exon 9 in the alternative splicing of the *Trp53bp1* gene, which is vital for maintaining genome stability (e.g., DNA double-strand break repair and chromosome synapsis) [43]. Intriguingly, the function of hnRNPH1 itself is subject to regulation. Wang et al. [44] demonstrated that MACC1 binds to the GYR domain of hnRNPH1 via its SH3 domain. This interaction enhances hnRNPH1 binding to target pre-mRNAs such as *IRAK1*, preventing the production of a shorter splice isoform (IRAK1-S). This suggests that MACC1 influences the splicing outcomes of specific genes by regulating hnRNPH1 activity, which was observed in a lung adenocarcinoma model [44]. The mechanisms of hnRNPH1-mediated splicing regulation are further depicted in Figure 1c.

Furthermore, hnRNPH1 can exert regulatory control by competing with other splicing factors to bind to key regulatory sites on pre-mRNAs. In the regulation of certain genes, such as *PRMT5*, competitive binding between hnRNPH1 and the antagonistic factor SRSF3 has been identified as a key mechanism governing AS under specific conditions, such as following radiation exposure (e.g., in breast cancer cells) [31]. hnRNPH1 may also compete with splicing-promoting factors such as Sam68 for binding sites, thereby influencing the final splice site selection [45]. In some instances, hnRNPH1 binding alone is sufficient to alter splicing patterns, and mutations within its binding sites can directly lead to changes in splicing outcomes [46].

## 3. Role of hnRNPH1 in Disease Pathogenesis

### 3.1. Cancer

hnRNPH1 exerts complex and often pro-tumorigenic functions during the development and progression of numerous cancers. Its mechanisms involve the precise regulation of multiple aspects of RNA metabolism, including AS, mRNA stability, translation, and subcellular localization, which in turn affect key signaling pathways and cellular phenotypes.

hnRNPH1 governs the AS of critical genes through tissue-specific and context-dependent mechanisms, representing a key pathway that influences tumorigenesis. In ES (Ewing Sarcoma), hnRNPH1 regulates the splicing of the *EWSR1::FLI1* fusion gene, promoting the production of oncogenic transcripts. This involves binding to G-rich sequences within exon 8 of the *EWSR1* gene, potentially involving the recognition and resolution of G4 structures [15,28]. In gliomas, hnRNPH1 promotes tumor progression by regulating the splicing of the pseudogene *PRELID1P6*, leading to the activation of the Akt/mTOR signaling pathway [47]. Notably, the knockdown of *PRELID1P6* induces the nucleocytoplasmic translocation and subsequent degradation of hnRNPH1, suggesting a potential interplay between splicing regulation and hnRNPH1 protein stability and localization [47]. hnRNPH1 also mediates the AS of *TRF2*, influencing the downstream Akt/mTOR pathway [47]. In non-small cell lung cancer, hnRNPH1, regulated by MYC, mediates a splicing switch of *KHK* pre-mRNA from the KHK-C to the KHK-A isoform, promoting metabolic reprogramming to support tumor growth [48]. In lung adenocarcinoma, hnRNPH1 interacts with the MACC1 protein (via its GYR domain binding to the MACC1 SH3 domain) to prevent the skipping of exon 11 in the IRAK1 gene’s pre-mRNA, thereby promoting the production of the oncogenic IRAK1-L isoform, driving tumorigenesis [44]. Aberrant expression or functional alterations of hnRNPH1 also correlate with clinical prognosis. In mantle cell lymphoma, non-coding mutations leading to dysregulated splicing and increased protein expression are significantly correlated with poor prognosis [49]. However, hnRNPH1 is not universally pro-oncogenic. In rhabdomyosarcoma, hnRNPH1 suppresses tumor growth by regulating the AS of genes such as *CTNNB1* and *MDM4*, leading to G1-phase cell cycle arrest [50]. Of greater interest, in oral tongue squamous cell carcinoma, the high expression of hnRNPH1 (along with WDR81) was associated with a favorable prognosis. Genes frequently mutated or exhibiting altered methylation in favorable prognosis cases were enriched in the MAPK pathway, suggesting a potential protective role for hnRNPH1 in this specific cancer type [51]. These findings underscore the significant context-dependency and cancer type specificity of hnRNPH1.

Regarding mRNA stability and translational regulation, hnRNPH1 significantly affects mRNA half-life and subsequent protein synthesis. Studies on colorectal cancer (CRC) have shown that hnRNPH1 interacts with the E3 ubiquitin ligase TRIM25, influencing the stability of caspase-7 mRNA [52]. Specifically, TRIM25 functions primarily as an RNA-binding protein, directly binding caspase-7 mRNA via its PRY/SPRY domain to promote its degradation; this function is notably independent of its E3 ligase activity [52]. hnRNPH1 also binds to caspase-7 mRNA and appears to promote its degradation in a manner synergistic with TRIM25, as knockdown of either protein increases mRNA stability [52]. While interacting on the mRNA, TRIM25 may ubiquitinate hnRNPH1, potentially contributing to this synergistic regulation, although the exact role of this ubiquitination requires further study [52]. This complex interplay potentially contributes to apoptosis regulation and enhanced chemoresistance in CRC [52]. Furthermore, hnRNPH1 promotes cell proliferation in CRC by directly binding to and stabilizing the SGPL1 mRNA, thereby inhibiting p53 activation [53].

In nasopharyngeal carcinoma, hnRNPH1 promotes malignant progression by stabilizing *FLOT2* mRNA in a noncanonical, m6A-independent manner [54]. In ovarian cancer, hnRNPH1 promotes tumor cell proliferation, migration, and invasion by stabilizing the long non-coding RNA (lncRNA) *LINC00662*, which indirectly leads to GRP78 upregulation and activation of the p38 MAPK signaling pathway [55]. Regarding translational regulation, although direct evidence in cancer is limited, the roles of hnRNPH1 in other systems suggest several potential mechanisms. For instance, hnRNPH1 is a component of the Lin28a complex in mouse embryonic stem cells (mESCs) that regulates the binding of *Dnmt3a* mRNA, potentially modulating its translation. This suggests that hnRNPH1 employs similar mechanisms to regulate the translation of cancer-related genes [56].

Regarding drug sensitivity, aberrant hnRNPH1 expression or activity is closely correlated with tumor cell responses to therapeutics. In CRC, hnRNPH1 confers resistance to chemotherapeutic drugs, such as doxorubicin, potentially via TRIM25-mediated inhibition of apoptosis [52]. In CML, high hnRNPH1 expression correlates with disease progression and imatinib resistance via the negative regulation of PTPN6, maintenance of PI3K/AKT pathway activation, and the positive regulation of BCR-ABL expression. Conversely, hnRNPH1 knockdown significantly enhances the sensitivity of CML cells to imatinib [16]. These studies suggest that targeting hnRNPH1 is a potential strategy for overcoming resistance to therapy in specific cancers.

The function of hnRNPH1 is intimately linked to its subcellular localization and extensive interaction networks. While predominantly found in the nucleus under normal conditions, its localization can be regulated by various factors. For instance, *PRELID1P6* knockdown can induce the nucleocytoplasmic translocation of hnRNPH1 and its subsequent degradation [47,57]. hnRNPH1 interacts with diverse proteins, including transcription factors (e.g., p53) [58], signaling molecules (e.g., MACC1) [59], and other RNA metabolism-associated proteins (e.g., TRIM25 [52], Lin28a, and their complex members) [56]. These interactions form the basis for the diverse regulatory functions of hnRNPH1. Pathway enrichment analyses have further implicated hnRNPH1 as a pro-tumorigenic node [21].

### 3.2. Neurological Disorders

hnRNPH1 plays critical roles in neurodevelopment by precisely regulating RNA splicing and stability, thereby ensuring the proper execution of neuronal differentiation programs. By recognizing G-rich RNA sequences, hnRNPH1 dynamically controls the splicing of neuron-specific exons. Aberrant hnRNPH1 expression or function can lead to neurodevelopmental defects, including autism spectrum disorder (ASD) [25,38]. Using an induced pluripotent stem cell model derived from patients with Rubinstein–Taybi syndrome, it was found that hnRNPH1 downregulation interferes with SRRM4-mediated neuronal-specific microexon splicing, leading to defects in neuronal differentiation and function. This suggests a pathological link between defects in chromatin regulation (e.g., mutations in *CBP/EP300* encoding p300) and aberrant RNA processing as a potential central mechanism underlying cognitive impairment [25]. Furthermore, a specific mutation (p.Arg206Trp) affecting the NLS of hnRNPH1 disrupts its nucleocytoplasmic shuttling equilibrium. This, in turn, inhibits the expression of pro-differentiation factors like TRF2-s, providing a molecular basis for the phenotypic diversity observed in hnRNPH1-related neurodevelopmental disorders [25].

Gillentine et al. [20] revealed that hnRNPH1 mutations contribute to neuropathology via a dual mechanism. First, mutations can directly impair RNA-binding capacity, disrupting RNA localization and local translation relevant to the neuronal differentiation of radial glial precursors. Second, mutations can disrupt the interaction networks with other RNA-processing proteins, impairing myelin protein synthesis [20]. Clinical data analysis showed that patients with these mutations exhibited characteristic structural brain abnormalities, such as microcephaly and cerebellar vermis hypoplasia, which correlated with high hnRNPH1 expression patterns in the striatum and amygdala. Phenotypic overlap with disorders caused by mutations in other *hnRNP* genes suggests functional synergy within this protein family [20]. These findings suggest that interventions targeting the aberrant RNA metabolic network downstream of hnRNPH1 represent a new therapeutic direction. For instance, restoring the activity of lysine acetyltransferases using epigenetic drugs might compensate for functional defects [20,60].

In the context of drug sensitivity and addictive behavior, hnRNPH1 regulates responses to multiple abused substances via the dynamic control of RNA splicing. Focusing on calcium channel regulation, Ruan et al. [61] demonstrated that hnRNPH1 alters the differential 3′ untranslated region (UTR) usage of *Cacna2d2* mRNA, a mechanism proposed to contribute to methamphetamine-induced dopamine release. Correspondingly, *Hnrnph1* mutant mice exhibit attenuated dopaminergic responses and reduced locomotor activity [61]. This effect was sex-specific, with female mutant mice showing more pronounced alterations in drug sensitivity, which was potentially linked to rapid adaptive changes in the hnRNPH1 targetome upon drug exposure [61]. Pharmacological studies confirmed this pathway: inhibition of the α2δ subunit (encoded by *Cacna2d2*) using pregabalin recapitulated the mutant phenotype in wild-type mice, which also showed increased usage of the proximal *Cacna2d2* 3′ UTR after drug treatment. This revealed a molecular pathway where hnRNPH1 influences neurotransmitter release by impacting *Cacna2d2*’s role in mediating the localization and function of voltage-gated calcium channel subunits [61].

The regulatory role of hnRNPH1 extends to opioids. Bryant et al. found that a heterozygous *Hnrnph1* mutation resulted in enhanced fentanyl reward effects in male mice, as evidenced by an increased conditioned place preference and operant response, whereas female mice showed the opposite tendency of diminished locomotor sensitization [62]. This sexual dimorphism contrasts with the female-predominant effects observed in the methamphetamine model, suggesting that hnRNPH1 might differentially regulate the interplay between dopaminergic and μ-opioid receptor systems via distinct RNA splicing pathways. Alcohol addiction models have broadened the functional spectrum of hnRNPH1. Fultz et al. [63] demonstrated that hnRNPH1 deletion not only reduced the voluntary intake of high-concentration alcohol in male mice but also reversed high-dose alcohol-induced conditioned place aversion. This switch in affective valence correlated with altered post-transcriptional processing of receptor genes such as *Oprm1* (encoding the μ-opioid receptor) [63]. In summary, how hnRNPH1 regulates addictive behaviors appears multifaceted, characterized by (1) significant effects on diverse classes of psychoactive substances (stimulants, opioids, alcohol), (2) multi-targeted regulation via different mechanisms (e.g., calcium channel localization and receptor processing), and (3) sex-dependent phenotypic outcomes, potentially influenced by the compensatory regulation of X-linked homologs such as hnRNPH2 [61,62,63]. These findings collectively outline the role of hnRNPH1 as an RNA metabolism hub within addiction circuitry. Its ability to rapidly remodel RNA-binding patterns in response to drug stimuli is a novel target for the development of broad-spectrum anti-addiction strategies based on splicing modulation.

In neurodegenerative diseases, hnRNPH1 dysfunction shares pathological parallels with other RBPs implicated in these conditions, such as TDP-43 and FUS, which are involved in RNA metabolism disturbances and abnormal protein aggregation [64]. hnRNPH1 participates in regulating RNA splicing and protein homeostasis through interactions with other molecular partners, such as SFPQ. Its aberrant function may exacerbate splicing defects and proteotoxic stress, which are characteristic of neurodegeneration, thereby accelerating neuronal injury [65]. Although the precise mechanisms remain unclear, studies suggest that hnRNPH1 plays a key role in maintaining neuronal RNA homeostasis, and its cancer-related pro-survival functions might paradoxically contribute to the pathology of neurodegeneration [21]. Additionally, hnRNPH1 has been linked to oligodendrocyte dysfunction in amyotrophic lateral sclerosis/frontotemporal lobar degeneration (ALS/FTLD) spectrum disorders, suggesting its involvement in disease processes via its effects on myelination [64].

Recent studies have also revealed the potential neuroprotective role of hnRNPH1 in neurodegeneration. Lin et al. found that hnRNPH1 was involved in neurodegenerative processes by regulating the expression of the neuroprotective cold-shock protein RBM3 [39]. hnRNPH1 binds to G-rich motifs within *RBM3* pre-mRNA, inhibiting the inclusion (retention) of a “poison exon”. This prevents the NMD of mRNA and enhances RBM3 protein expression [39]. This temperature-dependent regulatory mechanism not only provides a molecular basis for hypothermia-induced neuroprotection but also suggests that hnRNPH1 helps maintain neuronal homeostasis by dynamically responding to environmental stimuli. This regulation of *RBM3* is closely linked to RNA G-quadruplex (rG4) structures; disrupting rG4s significantly enhances poison exon retention, providing a rationale for developing rG4-targeting neuroprotective strategies [39]. In pathological states, hnRNPH1 mutations or functional abnormalities can lead to impaired RBM3 expression, weakening neuronal defenses against degenerative insults, a phenomenon with mechanistic similarities to the splicing dysregulation triggered by hnRNPH1 defects in neurodevelopmental disorders [39]. Enhancing hnRNPH1 activity or bypassing its regulatory step, for instance, through antisense oligonucleotides (ASOs) that inhibit poison exon inclusion or by mimicking hypothermic effects, could represent novel therapeutic directions for neurodegenerative disorders such as Alzheimer’s disease. This is particularly relevant given that RBM3 inhibits aberrant tau phosphorylation and synaptic dysfunction [39]. In summary, hnRNPH1 appears to play a dual role in neurodegenerative diseases, and its dysfunction can exacerbate neuronal injury through RNA dysmetabolism and proteotoxicity, while also exhibiting neuroprotective potential via the regulation of factors such as RBM3.

### 3.3. Reproductive System Disorders

hnRNPH1 plays multifaceted and critical roles in the development and functional regulation of the reproductive system, influencing germ cell development, gamete formation, and reproductive system homeostasis by regulating AS and transcriptional networks [19,66]. Specifically, during germ cell development, hnRNPH1 precisely regulates the AS of meiosis-associated genes by forming complexes with splicing factors, including PTBP2 and SRSF3. This includes regulating the ratio of *SPO11*α and *SPO11*β isoforms, directly participating in molecular processes like sex chromosome pairing and DNA damage repair [19,66]. The precision of splicing control is essential for maintaining germ cell transcriptome integrity; aberrations in this process can lead to impaired chromosome synapsis and germ cell apoptosis [19].

In the male reproductive system, hnRNPH1 maintains the spermatogenic microenvironment through a dual mechanism. On the one hand, it collaborates with PTBP1 to regulate the AS of genes crucial for Sertoli cell adhesion (e.g., *Espn* and *Pard6a*), safeguarding the structural integrity and selective permeability of the blood–testis barrier (BTB) [66]. In contrast, through interaction with the androgen receptor (AR), hnRNPH1 participates in regulating the transcriptional activation of genes within the EGFR signaling pathway, influencing the efficiency of communication between germ cells and Sertoli cells [66]. Disruption of these regulatory functions leads to delayed meiosis, premature sloughing of round spermatids, testicular atrophy, and, ultimately, male infertility [19,66]. The observation of BTB disruption (“cavitation”) following hnRNPH1 deletion suggests a potential spatiotemporally specific role in the dynamic remodeling of intercellular junction complexes, opening new avenues for investigating the mechanisms underlying male infertility.

Furthermore, hnRNPH1 exhibits distinct regulatory functions during oocyte development in the female reproductive system. It regulates the AS of genes such as *Brme1* and *Cpeb1* through synergistic interactions with SRSF3, influencing oocyte meiotic progression and the expression of cell junction proteins [19]. Feng et al. revealed that hnRNPH1 deletion leads to follicular developmental arrest at the primary follicle stage, accompanied by impaired communication between granulosa cells and oocytes, a phenotype highly reminiscent of clinical primary ovarian insufficiency [19]. hnRNPH1 participates in regulating intercellular communication in both the male and female reproductive systems yet exhibits sexual dimorphism regarding its specific targets and downstream molecular pathways. This suggests that hnRNPH1 maintains sex-specific reproductive functions through distinct AS regulatory networks.

hnRNPH1 dysfunction is also closely associated with various reproductive pathologies. In males, AR dysregulation may contribute to spermatogenic arrest and, potentially, azoospermia, whereas splicing abnormalities affecting the AR signaling pathway may be implicated in testicular dysgenesis syndrome [19,66]. In females, disruption of the hnRNPH1 regulatory network is associated not only with premature ovarian failure (related to oocyte depletion) but may also contribute to the pathogenesis of polycystic ovary syndrome by impacting the Wnt/β-catenin signaling pathway [19]. Although current studies have elucidated the fundamental regulatory mechanisms, hnRNPH1’s spatiotemporally specific functions within distinct germ cell subpopulations, crosstalk with epigenetic regulatory networks, and mutational landscapes in human reproductive disorders require further in-depth exploration. Future investigations employing single-cell multiomics technologies could dissect the dynamic hnRNPH1 network, potentially revealing novel targets for the precise diagnosis and treatment of reproductive disorders [19,66].

### 3.4. hnRNPH1 in Non-Cancer Pathologies

hnRNPH1 also has been implicated in diverse non-cancer pathologies, including muscle disorders, cardiovascular diseases, ocular disorders, autoimmune conditions, and viral hepatitis, and its regulatory mechanisms likely involve complex molecular networks and signaling pathways.

hnRNPH1 has been implicated in muscle disorders, particularly in the pathogenesis of Myotonic Dystrophy type 1 (DM1), a multisystemic disorder affecting skeletal muscle and characterized by aberrant RNA splicing [67,68]. In DM1 myoblasts, the steady-state levels of the splice regulator hnRNPH1 are found to be elevated, similar to those of CUG-BP1 [69]. This elevated hnRNPH1 contributes significantly to the characteristic aberrant splicing observed in DM1 cells, specifically impacting the alternative splicing of target RNAs like the Insulin Receptor (IR) exon 11, thereby recapitulating the splicing defect seen in DM1 [69]. Mechanistically, increased levels of either hnRNPH1 or CUG-BP1 can lead to the formation of an RNA-dependent suppressor complex involving both proteins, which is required for maximal repression of IR exon 11 inclusion [69]. Furthermore, hnRNPH1 also interacts with Muscleblind-like 1 (MBNL1), a key facilitator of IR exon 11 splicing and a protein functionally inactivated in DM1 due to sequestration by expanded repeats, in an RNA-independent manner [69]. This coordinated interplay among hnRNPH1, CUG-BP1, and MBNL1 is critical for regulating alternative splicing in muscle cells, and its dysregulation is a key driver of the aberrant splicing and resulting muscle and systemic dysfunction observed in DM1.

In the cardiovascular system, hnRNPH1 influences the expression of cardiovascular disease-related genes by recognizing G-rich RNA sequences and regulating RNA splicing and stability [38]. hnRNPH1 regulates vascular endothelial cell function and atherosclerosis progression. Furthermore, it promotes pathological cardiac hypertrophy during cardiac hypertrophy and heart failure by regulating cardiomyocyte RNA metabolism [70]. The role of hnRNPH1 is particularly notable in pulmonary arterial hypertension (PAH). Weighted gene co-expression network analysis and protein–protein interaction network analyses identified *hnRNPH1* as a core gene within the PAH-associated module, indicating its potential hub role in this disease [71]. Under hypoxic conditions that simulate PAH pathology, hnRNPH1 expression was significantly upregulated in pulmonary artery smooth muscle cells (PASMCs), suggesting its involvement in PAH pathogenesis [71]. Studies indicate that hnRNPH1 may contribute to the pathological process by regulating cell proliferation-related pathways, such as promoting the abnormal proliferation of PASMCs, an important feature of PAH [71]. hnRNPH1 also demonstrated potential as a diagnostic marker for PAH, with an area under the receiver operating characteristic curve of 0.744, indicating a good diagnostic discriminatory ability [71].

In ocular disorders, genetic variants of HNRNPH1 are associated with early onset high myopia in humans, with loss-of-function variants being significantly enriched among patients [72]. Zebrafish models further validated this link, as the knockdown of *hnrnph1* resulted in ocular defects and microphthalmia, suggesting that hnRNPH1 is essential for optic fissure closure and retinal development [72]. hnRNPH1 may also play a protective role during ocular stress by regulating the splicing of the cold-shock protein RBM3. This mechanism involves promoting the exclusion of a “poison exon” within the *RBM3* pre-mRNA [39]. In high-altitude retinopathy, hnRNPH1 expression is significantly downregulated, potentially participating in the cellular adaptive response to hypoxia by regulating RNA metabolism and transcription, further suggesting its value as a diagnostic marker [73].

hnRNPH1 functions as an autoantigen in conditions such as systemic lupus erythematosus and rheumatoid arthritis (RA), and the presence of autoantibodies against hnRNPH1 may correlate with disease onset and progression [13]. In an experimental autoimmune encephalomyelitis model of multiple sclerosis, the deletion of hnRNPH1 significantly inhibited Th17 cell differentiation and migration, attenuating neuroinflammation [13]. In RA, hnRNPH1 expression is upregulated in the synovial tissues and may contribute to disease progression by regulating synovial proliferation, inflammation, and fibrosis. Synergistic effects with other hnRNP family members (e.g., hnRNP-DL and hnRNP-K) also suggest their potential diagnostic and therapeutic value in RA [22,74]. In multiple sclerosis, aberrant hnRNPH1 expression may affect immune cell function and neuroinflammatory responses by regulating the AS of key genes such as *IL7R* and *SP140*. Its interaction with *MALAT1* further highlights its complex regulatory role in MS pathology [75].

**Figure 2:**
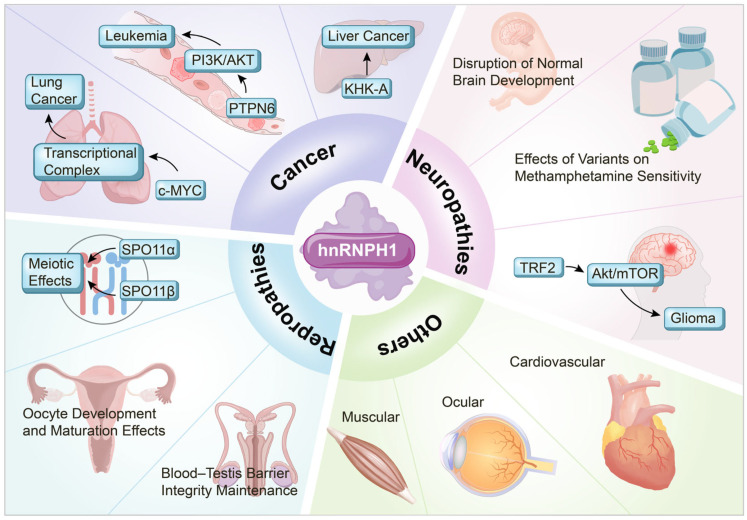
Multiple roles of hnRNPH1 in pathological and physiological processes.

hnRNPH1 has also been identified as an interacting partner of the hepatitis C virus (HCV) core protein HCVc174. This direct interaction is RNA-independent, suggesting that hnRNPH1 regulates viral replication through protein–protein binding [76]. During HCV infection, hnRNPH1 influences HCV replication and viral protein expression by modulating the host mRNA metabolism and viral RNA transport. Its nucleocytoplasmic shuttling properties may also be involved in the nucleocytoplasmic transport of HCVc174, thereby affecting both viral replication and host cell function [76]. In addition, the interaction between hnRNPH1 and HCVc174 may play a role in the development of chronic hepatitis and hepatocellular carcinoma, potentially promoting the malignant transformation of hepatocytes by altering host gene expression or RNA metabolism [76]. The multiple roles of hnRNPH1 in various pathological and physiological processes are summarized in Figure 2.

In cancer, hnRNPH1 contributes to leukemia progression through PI3K/AKT pathway modulation and PTPN6 regulation. It is involved in lung cancer progression, potentially linked to c-MYC activity, and impacts cancer cell metabolism partly through KHK-A splicing. In neurological roles, hnRNPH1 impacts normal brain development, modulates methamphetamine sensitivity (associated with genetic variants), and promotes glioma development via TRF2 splicing and Akt/mTOR pathway activation. hnRNPH1 plays crucial roles in reproductive processes, including meiotic regulation through SPO11α/β splicing, oocyte development and maturation, and maintenance of blood–testis barrier integrity. In other systems, hnRNPH1 influences muscle disorders, cardiovascular function, ocular development, and other pathologies, though the specific mechanisms governing these roles require further elucidation.

## 4. Therapeutic Implications

### 4.1. Current Therapeutic Strategies

As an important member of the RBP family, hnRNPH1 plays crucial roles in RNA splicing, gene expression regulation, and the pathogenesis of various diseases. In recent years, significant progress has been made in the development of therapeutic strategies targeting hnRNPH1. These include small-molecule inhibitors, gene silencing technologies, approaches targeting post-translational modifications (PTMs), and RNA-targeted therapies. These strategies target and alter hnRNPH1 function through diverse molecular mechanisms, offering novel therapeutic avenues and validating hnRNPH1 as a target for treating associated diseases.

Small-molecule inhibitors show remarkable potential for blocking interactions between hnRNPH1 and RNA. By targeting RNA recognition domains (RBDs) or interfering with RNA interactions, these inhibitors can effectively modulate gene expression and have shown promising applications in cancer therapy. Compounds 2155-14 and 2155-18 have been shown to bind and downregulate hnRNPH1, inducing apoptosis in melanoma cells while exhibiting low toxicity and a favorable therapeutic index in in vivo models [77]. Their mechanism involves the induction of endoplasmic reticulum stress and autophagy via the inhibition of hnRNPH1/H2, leading to antitumor effects [69]. Research targeting other RBPs provides valuable precedents for developing hnRNPH1-targeted therapies [78,79]. For instance, ebselen blocks the binding of YTHDF1 to m6A-modified RNAs by competitively binding to the YTHDF1 m6A recognition domain, demonstrating the potential of small-molecule inhibitors in regulating RBP function [78].

Gene silencing technologies, such as RNA interference (RNAi), offer high efficiency and specificity for knocking down hnRNPH1. Liu et al. demonstrated that the short hairpin RNA (shRNA)-mediated knockdown of hnRNPH1 significantly inhibited the proliferation of CML cells and enhanced their sensitivity to imatinib [16]. hnRNPH1 knockdown also significantly inhibited tumor growth in a nude mouse xenograft model [16]. Although RNAi faces challenges such as potential degradation by RNases and inefficient delivery, its therapeutic potential has been significantly improved by optimizing chemical modifications and delivery systems for small interfering RNAs (siRNAs) [80,81]. Chemically modified siRNAs with enhanced stability and target affinity can achieve durable gene silencing effects in vivo [80].

The PTMs of hnRNPH1 play a crucial role in regulating its function. For instance, phosphorylation can regulate RBP localization within cellular stress granules by modulating the LLPS behavior [82]. Moreover, arginine methylation is important for regulating the RBP-RNA-binding capacity and function [83]. For example, arginine methylation of METTL14 enhances its interaction with WTAP, promoting RNA m6A modification and influencing embryonic stem cell fate [83]. By developing inhibitors targeting these modifications (e.g., SRPK1 inhibitors), it is possible to effectively modulate hnRNPH1 function and show the potential for treating tumors and neurodegenerative diseases [82,84].

**Figure 3:**
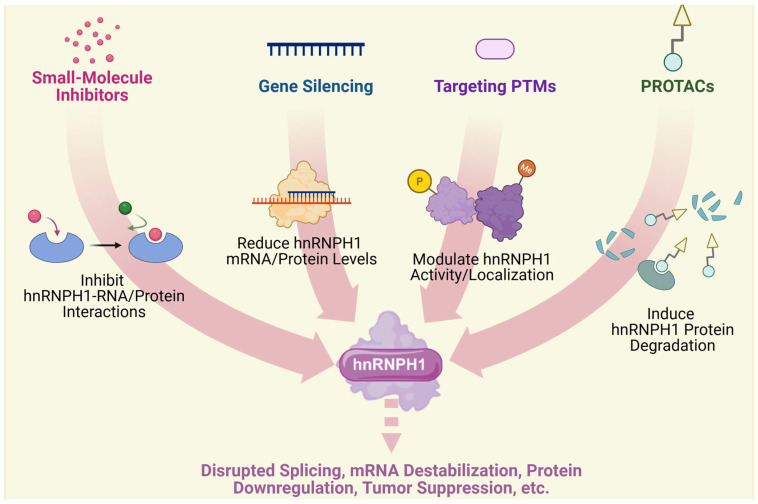
Therapeutic Strategies Targeting hnRNPH1. Schematic overview illustrating potential approaches to modulate hnRNPH1 function for therapeutic purposes: Small-Molecule Inhibitors target hnRNPH1 binding sites to inhibit its interactions with RNA or proteins. Gene Silencing methods (siRNA/ASO) reduce hnRNPH1 protein expression by targeting its mRNA or block hnRNPH1 RNA binding. Modulating hnRNPH1 PTMs affects its activity, stability, or localization. PROTACs induce specific hnRNPH1 protein degradation. These targeting strategies lead to downstream functional impacts, including disrupted splicing, mRNA destabilization, protein downregulation of target molecules, and effects on cellular processes such as tumor suppression, among other biological consequences.

ASOs and RNA aptamers are another class of therapeutics that can significantly interfere with hnRNPH1 binding to target RNAs. For example, L-aptamer-ASO conjugates can regulate gene expression by specifically recognizing rG4 structures and blocking the binding of helicases such as DHX36 to target RNAs [85]. These conjugates leverage both structural (aptamer) and sequence (ASO) recognition to enhance the binding affinity and specificity for their target rG4 regions [85]. Moreover, chemically modified ASOs, such as peptide nucleic acids, exhibit enhanced cell permeability and target affinity, leading to high efficiency in antiviral applications and gene silencing [86]. These studies demonstrate the broad applicability of ASOs and RNA aptamers in interfering with RBP function and offer promising tools and strategies for developing RNA-targeted therapies against hnRNPH1. Figure 3 provides a schematic representation of the therapeutic strategies discussed in this section and their resulting functional impacts.

### 4.2. Challenges and Optimizing Strategies

Although multiple therapeutic strategies targeting hnRNPH1 have shown significant potential in basic research and preclinical studies, their practical applications face numerous challenges. These hurdles primarily stem from the complexity and dynamic nature of hnRNPH1-RNA interactions, inefficient delivery, potential off-target effects associated with gene silencing technologies, the dynamic complexity of PTMs, and the inherent instability of RNA-targeted therapies. To overcome these barriers, optimization strategies, such as structure-based drug design (SBDD), advanced nanodelivery systems, specific enzyme inhibitors, chemical modification techniques, and targeted protein degradation, have been developed and explored.

The complexity and dynamic nature of RBP-RNA interactions represent major obstacles to the development of small-molecule inhibitors. RBPs typically engage RNA through multiple RBDs, involving extensive electrostatic, hydrogen bonding, and hydrophobic interactions, which makes the rational design of specific small-molecule inhibitors technically demanding [87,88]. The high abundance and functional diversity of RBPs further complicate the achievement of inhibitor selectivity, potentially leading to off-target effects or cytotoxicity [88]. SBDD and fragment-based screening have emerged as key strategies to address these challenges. For instance, by resolving the high-resolution structure of the N-terminal domain of the SARS-CoV-2 N protein complexed with RNA, Sarma et al. identified potential small-molecule inhibitors (ZINC00003118440 and ZINC0000146942) capable of specifically binding the RNA-binding cleft and inhibiting viral replication [89]. Similarly, fluorescence polarization-based screening for inhibitors of the LIN28-let-7 microRNA interaction identified trisubstituted pyrrolidinones that effectively disrupted PRI, modulating its biological function [79]. These studies highlight the critical role of structural elucidation in identifying druggable pockets within RBDs and guiding inhibitor design. Furthermore, high-throughput screening and chemical modification strategies offer important avenues for improving the permeability and stability of inhibitor cells. Wu et al. demonstrated that the inhibitor 1c, which targets HuR, directly binds its RNA-binding pocket, inhibits HuR function, and exhibits significant antitumor activity [90]. The combination of these approaches is expected to accelerate the development of potent and specific hnRNPH1 inhibitors and provide new tools for treating various diseases.

The major obstacles gene silencing technologies face in targeting RBPs are inefficient delivery and lack of specificity. Given that RBPs play pivotal roles in numerous essential cellular processes, broad gene silencing can lead to widespread cytotoxicity or off-target effects [91]. The high expression level of RBPs also makes it challenging to knock down their expression completely [91]. Nanodelivery systems and CRISPR/Cas9 gene editing technology offer potential solutions to address these problems. Fan et al. utilized a polycatechol-mediated siRNA delivery system in which multiple catechol moieties enhanced siRNA binding capacity and biostability, improving gene silencing efficiency in vivo [92]. Supe et al. developed liposome–polyethyleneimine complexes that protect siRNAs from nuclease degradation, demonstrating good serum stability and efficient HuR gene silencing in a diabetic retinopathy model, significantly reducing VEGF protein expression [93]. Additionally, the surface modification of nanoparticles can enhance targeted siRNA delivery. For example, a G-quadruplex proximity aptamer system targeting the transferrin receptor achieved efficient transmembrane siRNA transport and improved gene silencing [94]. CRISPR/Cas9 technology enables precise gene editing, effectively targeting RBP genes while minimizing off-target effects [91]. Using high-fidelity Cas9 variants (e.g., SpCas9-HF1) and optimizing the single-guide RNA (sgRNA) design significantly improves editing accuracy and specificity [95,96]. Integrating artificial intelligence and machine learning can further refine sgRNA prediction and optimization, reducing off-target risks [97]. Nevertheless, complex regulatory networks involving RBPs may complicate editing outcomes, thus requiring a deeper investigation of their functional mechanisms to optimize editing efficiency [98].

Targeting RBP PTMs is challenging because of their diversity and complexity. RBP PTMs are often dynamic and reversible and involve multiple enzymatic systems that complicate precise regulation [99]. Currently, the functions of many PTMs remain poorly understood, hindering the development of targeted inhibitors [99]. The development of specific enzyme inhibitors (e.g., kinase or deacetylase inhibitors) and the use of chemical probes are key strategies to address the complexities of targeting RBP PTMs. Inhibitors can precisely modulate the RBP modification status, while probes allow the real-time monitoring of modification sites, aiding drug development [99]. Proteolysis-targeting chimera (PROTAC) technology offers a novel therapeutic paradigm by hijacking the ubiquitin–proteasome system to induce the selective degradation of target proteins, including RBPs, in a modification-state-dependent manner [99]. PROTACs consist of a ligand for the target protein and an E3 ubiquitin ligase connected by a linker to form a ternary complex that triggers target ubiquitination and degradation [100,101]. Unlike the occupancy-driven mechanism of traditional inhibitors, PROTACs can achieve complete elimination of the target protein function [102,103]. For example, PROTACs targeting the Androgen Receptor (AR) demonstrated improved efficacy and overcame resistance by degrading the AR protein rather than merely inhibiting its activity [104]. PROTACs can also achieve selectivity by targeting noncatalytic domains, thereby expanding the targetable proteome. Studies targeting Janus kinases (JAKs) have shown that PROTACs can selectively degrade specific JAK family members, which is challenging for conventional inhibitors due to the high homology in their ATP-binding sites [105]. The design flexibility of PROTACs allows for the optimization of selectivity and degradation efficiency by modifying the linker and E3 ligase ligand. ARV-825, a BRD4 PROTAC, exhibits significant antitumor activity in various models (neuroblastoma, thyroid cancer, and T-ALL) by degrading the BRD4 protein and suppressing MYC expression, thereby inhibiting tumor cell proliferation [106,107,108]. In contrast, conventional BRD4 inhibitors like JQ1 are less effective against BRD4 overexpression and do not induce sustained protein degradation [109,110]. This degradation mechanism represents a promising strategy for overcoming drug resistance in hnRNPH1-associated diseases.

RNA-targeted therapies, such as ASOs and RNA aptamers, also face several challenges, including instability and inefficient delivery when targeting RBPs. The complex secondary and tertiary structures of RBP-RNA interactions can affect the binding efficiency of the ASO or aptamer [91,99]. ASOs and RNA aptamers are susceptible to in vivo nuclease degradation and require efficient delivery to the target cells [99]. Chemical modification is a core strategy for addressing these issues. Introducing modifications like phosphorothioate (PS) backbones or 2′-O-methyl (2′-OMe) sugars significantly enhances ASO stability and nuclease resistance [91,99]. PS modifications improve nuclease stability and protein binding, while 2′-OMe modifications increase thermal stability and target affinity [111,112]. Additionally, computer-aided design can optimize ASO or RNA aptamer secondary structures, further enhancing their binding affinity and specificity, paving the way for the precise design of RNA-targeted therapies [99].

## 5. Prospects

Despite the remarkable progress made in recent years regarding the structure, function, and disease roles of hnRNPH1, its complex regulatory networks and full biological significance as a multifunctional RBP remain incompletely understood, offering a vast scope for future exploration [113,114]. Future studies should focus on elucidating these intricate molecular mechanisms. This includes the in-depth characterization of the precise sequence and structural specificity of its RNA targets, particularly how their different domains cooperate and how conformational dynamics influence their functional selectivity [115,116,117]. Furthermore, systematically mapping the hnRNPH1 interactome under diverse physiological and pathological conditions and revealing how these interactions precisely govern its subcellular localization, phase separation behavior, and involvement in RNP granule assembly are crucial for understanding its complex regulatory roles [118,119]. Additionally, the specific regulatory mechanisms, dynamics, and functional impact of PTMs governing hnRNPH1 activity, such as phosphorylation and arginine methylation, warrant further investigation [120,121]. Clarifying the functional specificity versus potential redundancy between hnRNPH1 and its family homologs, especially in specific tissues and developmental stages, is also essential for a comprehensive understanding of its unique biological contributions [8,25,122].

Given the complex roles of hnRNPH1 in tumorigenesis, the maintenance of nervous system homeostasis, regulation of reproductive system development, and various other pathologies, future studies should aim to unravel its specific functions and associated molecular pathways within different disease contexts more deeply [123,124]. Defining the precise mechanisms by which hnRNPH1 promotes or inhibits specific disease types and stages is critical for evaluating its potential as a biomarker for the diagnosis, prognosis, or prediction of therapeutic response [125,126,127]. Particularly in cancer research, understanding the influence of hnRNPH1 on the tumor microenvironment, metabolic reprogramming, and the development of drug resistance, as well as in neurodegenerative diseases and developmental disorders, elucidating its impact on RNA metabolism, neuronal function, and protective mechanisms, will provide novel insights for deeper mechanistic understanding and the development of effective therapeutic interventions [117,128,129,130]. Moreover, the roles of hnRNPH1 in reproductive system disorders, cardiovascular diseases, and immune-related conditions merit further exploration, potentially yielding new targets and strategies for disease prevention and treatment [131,132,133].

Although the development of therapeutic strategies targeting hnRNPH1 is promising, significant challenges remain regarding its specificity, safety, and effective delivery [134,135,136,137]. Future efforts should leverage structural biology, medicinal chemistry, and computational modeling to design and screen more selective small-molecule inhibitors that could precisely target specific functional domains of hnRNPH1 or its disease-relevant interaction interfaces [138,139]. Optimizing gene silencing technologies and RNA-targeted therapies, for instance, by developing more effective chemical modifications and targeted delivery systems, is crucial for improving their in vivo stability and tissue specificity, particularly for overcoming physiological barriers such as the blood–brain barrier [138,140]. Moreover, exploring enzyme inhibitors or activators that target key PTMs regulating hnRNPH1, along with utilizing emerging technologies like PROTACs for the selective degradation of aberrant hnRNPH1 proteins, may offer new avenues to overcome drug resistance [6,141,142,143].

## 6. Conclusions

hnRNPH1 is a multifunctional RBP. By utilizing its unique structural domains, it recognizes and binds to G-rich RNA sequences and plays a central role in regulating multiple RNA metabolic processes, including AS, mRNA stability, and translation. These functions are essential for maintaining cellular homeostasis and the normal function of diverse physiological systems, notably the nervous and reproductive systems. Therefore, hnRNPH1 dysfunction is closely associated with a variety of human diseases. Its involvement includes a complex, often tumor-promoting, yet context-dependent role in various cancers; significant impacts on neurodevelopment and neurological homeostasis; contributions to reproductive disorders; and participation in various non-cancer pathologies, such as cardiovascular and autoimmune diseases. Given its broad pathophysiological significance, hnRNPH1 represents a promising therapeutic target. Current strategies, including small-molecule inhibitors, gene silencing techniques, and RNA-targeted therapies, have also demonstrated potential. However, they still face considerable challenges regarding specificity, delivery efficiency, and the emergence of drug resistance. Furthermore, novel technologies such as PROTACs offer promising avenues to overcome these obstacles. Future studies are still needed to elucidate the precise regulatory mechanisms and intricate functional networks governed by hnRNPH1. Efforts should also be focused on developing more precise and safer targeted therapeutic strategies than the ones currently available. The ultimate aim is to translate fundamental research findings into effective clinical interventions, thereby advancing the diagnosis and treatment of hnRNPH1-associated diseases.

## Data Availability

Data sharing is not applicable to this article as no new datasets were generated or analyzed during the preparation of this review. All information discussed relies on previously published literature cited in the references.

## Abbreviations

**Abbreviation**	**Full Name**
AI	Artificial Intelligence
ALS/FTLD	Amyotrophic Lateral Sclerosis/Frontotemporal Lobar Degeneration
AR	Androgen Receptor
AS	Alternative Splicing
ASD	Autism Spectrum Disorder
ASO	Antisense Oligonucleotide
AUC	Area Under the Curve
BBB	Blood–Brain Barrier
BCP-ALL	B-Cell Precursor Acute Lymphoblastic Leukemia
BTB	Blood–Testis Barrier
CML	Chronic Myeloid Leukemia
CPA	Conditioned Place Aversion
CPP	Conditioned Place Preference
CRC	Colorectal Cancer
DM1	Myotonic Dystrophy Type 1
EAE	Experimental Autoimmune Encephalomyelitis
eoHM	Early-Onset High Myopia
ER	Endoplasmic Reticulum
ES	Ewing Sarcoma
G4	G-quadruplex
G4PA	G-quadruplex Proximity Aptamer
G-rich	Guanine-rich
GY	Glycine-rich region
GYR	Glycine-Tyrosine-Arginine-rich region
HAR	High-Altitude Retinopathy
HCC	Hepatocellular Carcinoma
HCV	Hepatitis C Virus
hnRNP	Heterogeneous Nuclear Ribonucleoprotein
hnRNPH1	Heterogeneous Nuclear Ribonucleoprotein H1
HTS	High-Throughput Screening
iPSC	Induced Pluripotent Stem Cell
JAK	Janus Kinase
KAT	Lysine Acetyltransferase
LCD	Low-Complexity Domain
LLPS	Liquid–Liquid Phase Separation
lncRNA	Long Non-coding RNA
LoF	Loss-of-Function
MCL	Mantle Cell Lymphoma
mESC	Mouse Embryonic Stem Cell
ML	Machine Learning
MS	Multiple Sclerosis
NLS	Nuclear Localization Signal
NMD	Nonsense-Mediated Decay
NPC	Nasopharyngeal Carcinoma
NSCLC	Non-Small Cell Lung Cancer
NTD	N-Terminal Domain
OTSCC	Oral Tongue Squamous Cell Carcinoma
PAH	Pulmonary Arterial Hypertension
PASMC	Pulmonary Artery Smooth Muscle Cell
PCOS	Polycystic Ovary Syndrome
PNA	Peptide Nucleic Acid
POF	Premature Ovarian Failure
POI	Primary Ovarian Insufficiency
PPI	Protein–Protein Interaction
pre-mRNA	Precursor Messenger RNA
PRI	Protein-RNA Interaction
PROTAC	Proteolysis-Targeting Chimera
PS	Phosphorothioate
PTM	Post-Translational Modification
qRRM	Quasi-RNA Recognition Motif
RA	Rheumatoid Arthritis
RBP	RNA-Binding Protein
RBD	RNA Recognition Domain
rG4	RNA G-quadruplex
RNAi	RNA Interference
RNP	Ribonucleoprotein
ROC	Receiver Operating Characteristic
RRM	RNA Recognition Motif
RSTS	Rubinstein–Taybi Syndrome
SBDD	Structure-Based Drug Design
sgRNA	Single Guide RNA
shRNA	Short Hairpin RNA
siRNA	Small Interfering RNA
SLE	Systemic Lupus Erythematosus
TDS	Testicular Dysgenesis Syndrome
TfR	Transferrin Receptor
UTR	Untranslated Region
WGCNA	Weighted Gene Co-expression Network Analysis
WT	Wild-Type
2′-OMe	2′-O-methyl
